# Predicting the Effects of Carbapenem/Carbapenemase Inhibitor Combinations against KPC-Producing *Klebsiella pneumoniae* in Time-Kill Experiments: Alternative versus Traditional Approaches to MIC Determination

**DOI:** 10.3390/antibiotics10121520

**Published:** 2021-12-11

**Authors:** Alla V. Filimonova, Maria V. Golikova, Elena N. Strukova, Yury A. Portnoy, Anastasiya A. Kuznetsova, Stephen H. Zinner

**Affiliations:** 1 Department of Pharmacokinetics & Pharmacodynamics, Gause Institute of New Antibiotics, 11 Bolshaya Pirogovskaya Street, 119021 Moscow, Russia; allafil@yandex.ru (A.V.F.); lena-stru@inbox.ru (E.N.S.); yaportnoy@gmail.com (Y.A.P.); biolisichka@gmail.com (A.A.K.); 2 Department of Medicine, Harvard Medical School, Mount Auburn Hospital, 330 Mount Auburn St., Cambridge, MA 02138, USA; szinner@mah.harvard.edu

**Keywords:** β-lactams, β-lactamase inhibitors, imipenem, doripenem, relebactam, *Klebsiella pneumoniae*, time-kill assay

## Abstract

Traditionally, the antibacterial activity of β-lactam antibiotics in the presence of β-lactamase inhibitors is determined at the fixed inhibitor concentration. This traditional approach does not consider the ratio of antibiotic-to-inhibitor concentrations achieved in humans. To explore whether an alternative pharmacokinetically based approach to estimate MICs in combinations is predictive of antimicrobial efficacy, the effects of imipenem and doripenem alone and in combination with relebactam were studied in time-kill experiments against carbapenemase-producing *Klebsiella pneumoniae*. The carbapenem-to-relebactam concentration ratios in time-kill assays were equal to the therapeutic 24-h area under the concentration-time curve (AUC) ratios of the drugs (1.5/1). The simulated levels of carbapenem and relebactam were equal to their concentrations achieved in humans. When effects of combined regimens were plotted against respective C/MICs, a sigmoid relationship was obtained only with MICs determined by pharmacokinetically based method. The effectiveness of both carbapenems in the presence of relebactam was comparable by the results of time-kill experiments. These findings suggest that (1) antibiotic/inhibitor MICs determined at a pharmacokinetically based concentration ratio allow an adequate assessment of carbapenem susceptibility in carbapenemase-producing *K. pneumoniae* strains and can be used to predict antibacterial effects; (2) in time-kill experiments, the effects of imipenem and doripenem in the presence of relebactam are comparable.

## 1. Introduction

β-lactam/β-lactamase inhibitor combinations are useful options to combat increasing antimicrobial resistance among multidrug- resistant β-lactamase producing Gram-negative bacteria. The combination of imipenem with the novel β-lactamase inhibitor relebactam is effective against high-level carbapenem-resistant *Klebsiella pneumoniae* [1,2,3,4]. This combination was recently approved for clinical use in the treatment of hospital-acquired and ventilator-associated bacterial pneumonia, complicated urinary tract infections and complicated intra-abdominal infections [5,6,7]. Relebactam has a potent in vitro activity against class A β-lactamases, including *Klebsiella pneumoniae* carbapenemases (KPC-type) and class C β-lactamases [8,9].

As recommended by CLSI, the antibacterial activity of imipenem/relebactam, as estimated by the MIC (minimal inhibitory concentration), is usually determined by varying imipenem concentrations in the presence of a fixed concentration of relebactam [10]. However, this traditional approach to MIC determinations for antibiotic/inhibitor combinations might be inadequate, as it does not consider the actual antibiotic-to-inhibitor concentration ratios achieved in humans. The search for optimal predictors of clinical outcome and antibacterial effects remains important to optimize treatment. From this point of view, a previously described approach to determining MICs of antibiotics in combinations using pharmacokinetically based (PK-based) concentration ratios seems promising [11,12,13,14,15]. According to this PK-based approach, to predict antibacterial effects of antibiotics used in combination in an in vitro dynamic model by their MICs in the presence of each other, antibiotic-to-antibiotic concentration ratios during MIC determinations should be equal to respective antibiotic-to-antibiotic area under the concentration-time curve (AUC) ratios when simulated in pharmacodynamic experiments. The appropriateness of this approach to predict efficacy of combination therapies was confirmed in a series of in vitro pharmacodynamic studies with several antibiotic combinations and Gram-positive [11,12,13,14] and Gram-negative [15] bacteria. With regard to antibiotic/inhibitor combinations, a similar approach was used to study efficacy of the combination of a β-lactam antibiotic, ampicillin, with a β-lactamase inhibitor, sulbactam [16]. Using an in vitro dynamic model, a reasonable correlation was found between ampicillin MICs determined at a PK-based ampicillin-to-sulbactam concentration ratio and the antibacterial effect of ampicillin therapeutic dose in the presence of a sulbactam therapeutic dose against β-lactamase-producing *Escherichia coli*. In contrast, no correlation with the effect of ampicillin plus sulbactam was found when antibiotic MICs were determined at a fixed sulbactam concentration (traditional approach to MIC determination).

To explore whether this alternative, PK-based approach to antibiotic MIC estimation in the presence of inhibitor is predictive of the efficacy of carbapenem/carbapenemase inhibitor combinations, the antibacterial effects of imipenem and doripenem alone and in combination with relebactam were studied in time-kill experiments against high-level carbapenem-resistant KPC-producing *K. pneumoniae*. To date, the combination of doripenem with relebactam has not been studied extensively. It is worth noting that the present study was carried out in static conditions in contrast to previously cited studies in dynamic models [11,12,13,14,15,16]. We aim to validate the predictive potential of a PK-based approach to MIC estimation by the results of experiments conducted in the static conditions of time-kill assays, widely used to investigate the in vitro antibacterial activity of drugs. In all time-kill experiments, the simulated imipenem, doripenem and relebactam concentrations were equal to their concentrations achieved in humans (healthy adults) over the entire dosing interval (from peak to trough) and included the average steady-state concentrations. It worth noting, as both imipenem and doripenem characterize with similar elimination half-lives as relebactam, the drugs concentration ratio could be assumed to be constant throughout the entire dosing interval and equal to 1.5/1. Therefore, in all time-kill experiments this constant 1.5/1 concentration ratio was realized.

## 2. Results

### 2.1. MICs of Imipenem or Doripenem Alone and in the Presence of Relebactam

Using method 1, MICs of imipenem and doripenem in the presence of 4 mg/L relebactam were reduced 128-256-fold against both *K. pneumoniae* strains (Table 1). Using method 2, carbapenem susceptibilities of the tested *K. pneumoniae* strains in the presence of relebactam differed; depending on the strain, MICs were reduced 16-64 and 8-32-fold for imipenem and doripenem, respectively. Given the CLSI MIC breakpoints, MIC testing by method 1 yielded carbapenem “susceptibility” for both strains (we used the CLSI MIC breakpoint for doripenem alone as there is no reported MIC breakpoint for the doripenem/relebactam combination) [17]. However, MIC results with method 2 showed the clinical *K. pneumoniae* strain to be resistant to both imipenem and doripenem and the ATCC strain to be intermediately susceptible to doripenem and imipenem.

### 2.2. Time-Kill Experiments with K. Pneumoniae

**Figure 1:**
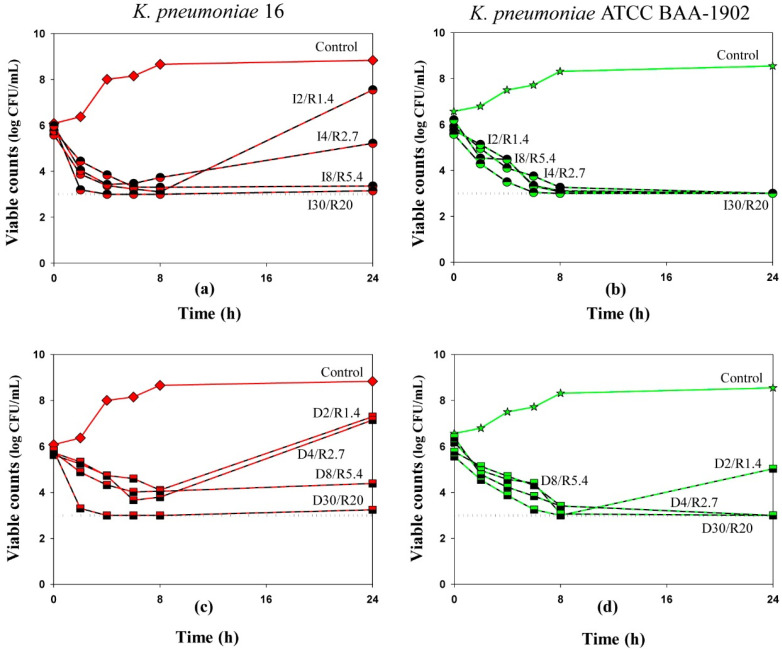
Time-kill curves of imipenem (**a**,**b**) and doripenem (**c**,**d**) used in combination with relebactam against *K. pneumoniae*. Dosing regimens are indicated at each curve. Dotted lines indicate the limit of detection. Data are presented as arithmetic means. Standard deviations are not shown as the data point difference was negligible.

When used alone, imipenem and doripenem reduced the initial bacterial numbers over the first 8 h in a concentration-dependent manner—the higher the carbapenem concentration, the lower the numbers of minimal viable counts (data shown in Supplementary Figure S1). The maximum reduction of bacterial counts (2 log CFU/mL for the clinical isolate; 2.5 log CFU/mL for the ATCC strain) was observed with regimens that contained 30 mg/L of imipenem or doripenem (equal to the therapeutic peak concentration). Following the initial reduction, regrowth was observed at 24 h of observation in all mono-exposure experiments. In the combination experiments during the first 8 h, imipenem/relebactam and doripenem/relebactam produced initial bacterial killing at least by 1.5 log CFU/mL or up to the limit of detection (Figure 1). However, as seen in the Figure, bacterial regrowth occurred by 24 h in experiments with *K. pneumoniae* 16 exposed to I2/R1.4, I4/R2.7, D2/R1.3 and D4/R2.7 and with *K. pneumoniae* ATCC 1902 exposed to D2/R1.4. These differences in bacterial killing among these carbapenem/inhibitor regimens could not be explained by the MICs determined by method 1 at the constant concentration of relebactam; for both strains, carbapenem concentrations were always at least 4-fold higher than the respective MICs. However, susceptibility of both *K. pneumoniae* strains to imipenem and doripenem in the presence of relebactam determined by method 2 was consistent with regrowth observed in time-kill experiments: regrowth occurred when carbapenem concentrations were equal to or lower than the respective MIC.

**Figure 2:**
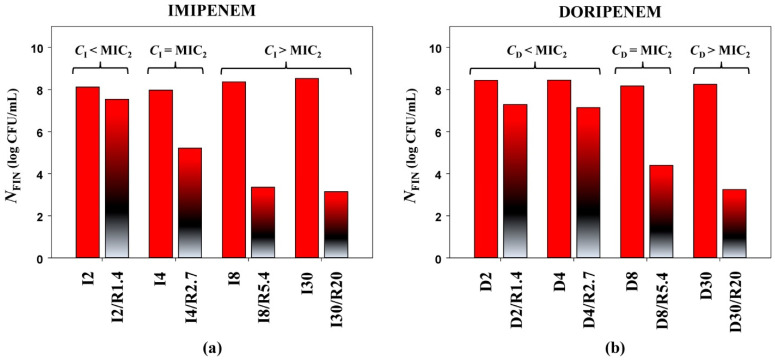
Antimicrobial effects of imipenem (**a**) and doripenem (**b**) (expressed as *N*_FIN_), alone and in combination with relebactam against *K. pneumoniae* 16 (MIC_2_—MIC obtained with the PK-based method).

This observation is demonstrated in Figure 2, which compares bacterial counts based on antibiotic concentrations alone or in combination with relebactam for the *K. pneumoniae* 16. As seen in the Figure, high bacterial counts at the end of the experiments (*N*_FIN_) were associated with concentrations of imipenem (Figure 2a) or doripenem (Figure 2b) equal to or lower than the carbapenem MIC as determined at PK-based carbapenem-to-relebactam concentration ratios (MIC_2_). When carbapenem concentration was higher than its MIC_2_, bacterial counts were close or equal to the lower limit of detection. These data suggest that MIC determinations at PK-based carbapenem-to-relebactam concentration ratios might be better in vitro predictors of antibacterial effects than MICs determined at a fixed concentration of relebactam, i.e., at an arbitrary antibiotic/inhibitor ratio.

**Figure 3:**
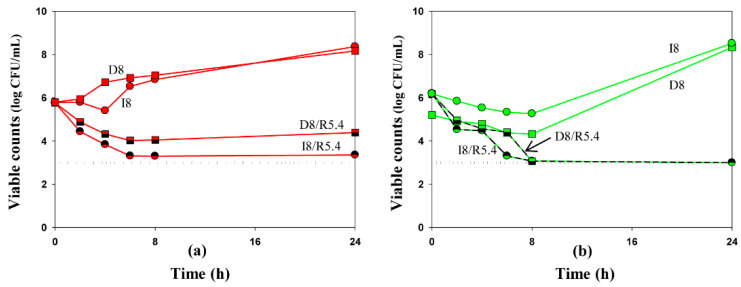
Time-kill curves at average steady-state concentrations of imipenem (circles) and doripenem (squares), alone and in combination with relebactam against *K. pneumoniae* 16 (**a**) and ATCC BAA-1902 (**b**). Dosing regimens are indicated at each curve. Dotted lines indicate the limit of detection. Data are presented as arithmetic means. Standard deviations are not shown as the data point difference was negligible.

To compare the antibacterial effectiveness of imipenem and doripenem alone or in the presence of relebactam, we grouped the time-kill curves by the same antibiotic concentration at average steady-state concentration as an example (Figure 3). As seen in the Figure, against the clinical *K. pneumoniae* strain, doripenem alone or in combination with relebactam was slightly less effective than imipenem. With the ATCC strain at 8 h, doripenem alone resulted in residual counts 1 log CFU/mL lower than imipenem; at 24 h regrowth was observed with both antibiotics. However, both carbapenems were similarly effective in combination with relebactam.

## 3. Discussion

Antimicrobial susceptibility testing is essential for predicting the clinical efficacy of antibacterial agents. As β-lactam/β-lactamase inhibitor combinations are widely used to treat seriously ill patients with hospital-acquired infections, reliable MIC estimations are critical in optimizing therapeutic regimens.

Currently MIC determination by varying antibiotic concentrations in the presence of a constant inhibitor concentration is the traditional approach to determining the susceptibility of pathogenic bacteria to β-lactam antibiotics in combination with β-lactamase inhibitors [10]. MICs determined by this method provide susceptibility estimations at arbitrary antibiotic/inhibitor concentration ratios, which does not consider the pharmacokinetic properties of the tested drugs. As such, this approach could provide an inadequate prediction of the antibacterial potential of an antibiotic/inhibitor combination. Thus, there is a need for alternative approaches to predict antibacterial effectiveness of antibiotic/inhibitor combinations.

Recently a PK-based approach to MIC determinations of antibiotic combinations was confirmed as reliable to predict antibacterial efficacy in in vitro pharmacodynamic experiments [11,12,13,14,15]. A similar approach effectively predicted the efficacy of ampicillin plus sulbactam [16]. The essence of this approach is as follows: to adequately predict the in vitro efficacy of antibiotic/inhibitor combination and its clinical relevance by MIC, the antibiotic-to-inhibitor concentration ratio in susceptibility testing should be equal to the ratio of therapeutic AUCs of these drugs.

In the present study, imipenem/relebactam or doripenem/relebactam combination efficacy was evaluated in time-kill experiments with high-level carbapenem-resistant *K. pneumoniae* strains. These data were used to distinguish between the predictive potential of PK-based and traditional approaches to MIC determination. The simulated in time-kill assays imipenem, doripenem and relebactam concentrations were equal to their concentrations achieved in humans over the entire dosing interval (from peak to trough) and included the average steady-state concentrations. The results of these experiments suggested a more accurate method for antibiotic/inhibitor combination MIC estimations.

The choice to conduct time-kill experiments that assess antimicrobial activity at static conditions was made because this methodology is widely used to evaluate in vitro antimicrobial effectiveness, including antibiotic combinations. However, the precise concentration ratios of antibiotics and inhibitors to use in time-kill experiments are not clearly understood. Most study designs use antimicrobial concentration as a multiple of the MIC, but the inhibitor is at constant concentration, similar to traditional MIC testing [18,19,20]. This approach does not consider antibiotic pharmacokinetics and therefore the actual antibiotic/inhibitor concentration ratios achieved in humans. We postulate that it would be preferable to use pharmacokinetically based drug concentrations and antibiotic/inhibitor ratios that are achievable in humans, as this allows a better assessment of the clinical relevance of the tested combination. For this reason, the study design provided the range of carbapenem and inhibitor concentrations achieved in humans at a fixed 1.5/1 ratio (from peak to trough over the entire dosing interval), including average steady-state levels. The average steady-state concentrations reflect the average exposure for each agent in combination over the 24-h experiment. Recently, a similar approach was applied in several time-kill studies with imipenem/relebactam [21] and ceftazidime/avibactam in which average steady-state concentrations were simulated [22].

In the current research in the presence of relebactam, regrowth of *K. pneumoniae* strains was observed at concentrations of imipenem and doripenem when carbapenem levels were equal to or lower than their MICs determined at a PK-based carbapenem-to-relebactam concentration ratio (MIC_2_). Imipenem and doripenem MICs estimated at a fixed relebactam concentration (MIC_1_) obviously were below the simulated antibiotic concentrations and thus could not explain the bacterial regrowth.

**Figure 4:**
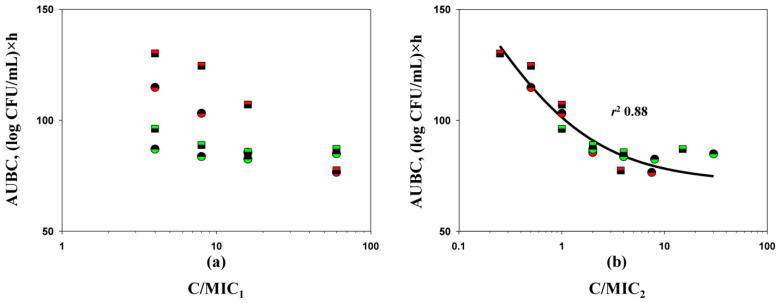
C/MIC_1_ (**a**) and C/MIC_2_ (**b**)-dependent antimicrobial effects (expressed as AUBCs) of imipenem (circles) and doripenem (squares) in combination with relebactam on *K. pneumoniae* 16 (red color) and *K. pneumoniae* ATCC BAA-1902 (green color) in time-kill experiments (MIC_1_—MIC_S_ obtained with the traditional method; MIC_2_—MIC_S_ obtained with the PK-based method). The relationship fits by Equation (1): *Y*_0_ = 72.00, *x*_0_ = 1.412, *a* = 155.4, *b* = −0.5838.

To more accurately discriminate between the predictive potential of MICs determined by the two methods, the correlation between antimicrobial effects (expressed as AUBC) observed in time-kill experiments and the concentration/MIC ratio (C/MIC, MIC determined by method 1 or method 2) was evaluated (merged data for both antibiotics and bacterial strains, Figure 4). A strong correlation between AUBC and C/MIC was observed when MIC data from the PK-based (method 2) carbapenem-to-relebactam concentration ratio were used (*r*^2^ 0.88). When MIC data by method 1 were used, a correlation between the effect and exposure was not observed. In another time-kill study with the imipenem/relebactam combination where average steady-state concentrations of drugs were simulated, the authors reported a consistent relation between the antimicrobial effect of the combination against *Pseudomonas aeruginosa* strains and MICs determined in the presence of a fixed inhibitor concentration [21]. However, the authors did not provide any quantitative analysis to support this observation, and they did not determine imipenem MICs in the presence of relebactam at PK-based concentration ratios to compare the predictive potential of these two MIC determination methods.

Based on the results of a previous study with ampicillin/sulbactam [16] and the current research, we believe that universal design of antibiotic/inhibitor combination MIC determinations and time-kill assays using antibiotic and inhibitor concentration ratios that reflect their ratios in humans could be a valuable option to assess the antibacterial effectiveness of combinations. In addition, we would like to point out that our findings with imipenem/relebactam and doripenem/relebactam combinations need to be confirmed in the dynamic conditions using an in vitro dynamic model. The pharmacokinetic–pharmacodynamic (PK/PD) modelling is a valuable option to measure the antibacterial efficacy of antimicrobials, including antibiotic/inhibitor combinations [23]. Compared to time-kill experiments, this allows more reliable description of the interaction between the drug and bacteria as antibiotic concentration change in accordance with that in human. Based on the results of PK-based MIC testing, PK-based time-kill assay and PK/PD modelling, it would be possible to create the comprehensive methodology to investigate in vitro effectiveness of beta-lactam/beta-lactamase combinations and adequately assess their clinical relevance.

Notably, the antibacterial effects of imipenem and doripenem in the presence of relebactam as determined in time-kill experiments were similar for the ATCC *K. pneumoniae* strain; imipenem was slightly more effective against the clinical isolate, although this difference was not statistically significant. These data indicate that imipenem and doripenem in combination with relebactam can provide similar antibacterial effects against *K. pneumoniae* strains. However, additional experiments are needed to support these results.

Our study has several limitations. It was performed as a proof-of-concept study to provide evidence of the applicability of a PK-based approach to MIC estimation; as such, it did not include many bacterial strains or other antibiotic/inhibitor combinations. This is our first attempt to validate the PK-based approach in time-kill experiments with carbapenem/carbapenemase inhibitor combinations; subsequent studies with a wider range of carbapenemase-producing pathogens and antibiotic/inhibitor combinations would enhance the generalizability of our results. Moreover, experiments in in vitro dynamic models are necessary to further validate the PK-based approach to these combinations.

## 4. Materials and Methods

### 4.1. Antimicrobial Agents and Bacterial Strains

Imipenem monohydrate and doripenem hydrate powders were purchased from Acros Organics (Fair Lawn, NJ, USA); relebactam was purchased from Invivochem (Libertyville, IL, USA). A clinical isolate, *K. pneumoniae* 16 and *K. pneumoniae* ATCC BAA-1902 were used in the study; *K. pneumoniae* ATCC 700603 was used as negative control. Carbapenemase production was confirmed for each bacterial strain by a modified carbapenem-inactivation method [24].

### 4.2. Susceptibility Testing

Susceptibility testing for antibiotics and inhibitor used alone or in combination was performed using broth microdilution techniques with inocula of approximately 5×10^5^ CFU/mL. When used alone, antibiotics were tested according to a standard broth microdilution methodology [10], while for combinations MIC testing was performed under two different conditions as determined by method 1 or method 2 regarding the ratio of imipenem or doripenem to relebactam. Before reading, plates were incubated at 37 °C for 18 h. MIC values were obtained in triplicate.

Method 1 (traditional, MIC_1_). MIC testing for imipenem/relebactam and doripenem/relebactam combinations used a fixed relebactam concentration of 4 mg/L with doubling dilutions of imipenem or doripenem according to CLSI recommendations.

Method 2 (PK-based, MIC_2_). MIC testing for imipenem/relebactam and doripenem/relebactam combinations used a fixed PK-based carbapenem-to-relebactam concentration ratio of 1.5/1 by varying the carbapenem and relebactam concentrations in parallel in each subsequent dilution. This concentration ratio is equal to the therapeutic 24-h AUC ratio of imipenem or doripenem (for a 500 mg dose of each carbapenem every 6 h [25,26]) to the therapeutic AUC of relebactam (for a 250 mg dose every 6 h [26]). The PK-based ratio was equal for imipenem/relebactam and doripenem/relebactam combinations as both carbapenems are characterized with similar pharmacokinetic profiles.

### 4.3. Time-Kill Assay Procedure

Time-kill assays were performed in duplicate with each *K. pneumoniae* strain. At the start of the experiment, Mueller-Hinton broth (MHB) was inoculated with a 24-h bacterial suspension to provide a final density of approximately 10^6^ CFU/mL. Time-kill experiments with imipenem or doripenem used alone or in combination with relebactam and control growth experiments were conducted. Tubes containing 20 mL of MHB with bacteria and antimicrobials alone or in combination with relebactam were incubated at 37 °C for 24 h. At 0, 2, 4, 6, 8 and 24 h the tubes were sampled to quantify the bacterial counts. Samples (100 µL) were serially diluted as appropriate and plated onto Mueller-Hinton agar plates, which were placed in an incubator at 37°C for 24 h. The lower limit of accurate detection was 1 × 10^3^ CFU/mL.

### 4.4. Drug Exposures

Relebactam content in all combination experiments with each carbapenem corresponded to a PK-based antibiotic-to-inhibitor ratio of 1.5/1. It was possible to use the same carbapenem-to-inhibitor concentration ratio for each time-point across the dosing interval as both imipenem and doripenem as well as relebactam are characterized with similar elimination half-lives. The concentrations of imipenem or doripenem (used alone or in combination with relebactam) and relebactam (only in combined experiments) in time-kill assays varied over a wide range and corresponded to levels achieved in humans over the entire dosing interval: 30, 8, 4, 2 mg/L for carbapenems and 20, 5.4, 2.7, 1.4 mg/L for relebactam [25,26]. Whereas 30 and 20 mg/L are peak plasma carbapenem and relebactam concentrations, respectively, 8 and 5.4 mg/L are average steady-state, and 2 and 1.4 mg/L are trough plasma carbapenem and relebactam concentrations, respectively. The average drug steady-state concentration was determined as the ratio of 24-h AUC of the drug to the 24-h time period.

Simulated carbapenem concentrations in mono-exposure experiments were as follows: imipenem 30, 8, 4 and 2 mg/L, designated as I30, I8, I4 and I2, respectively; doripenem 30, 8, 4 and 2 mg/L—D30, D8, D4 and D2, respectively.

The simulated carbapenem and relebactam concentrations in combined experiments were as follows: I30 plus relebactam (R) 20 mg/L designated as I30/R20, I8 plus R 5.4—I8/R5.4, I4 plus R 2.7—I4/R2.7 and I2 plus R 1.4—I2/R1.4; D30 plus R20—D30/R20 and other D/R regimens were designated in the same manner—D8/R5.4, D4/R2.7, D2/R1.4.

### 4.5. Quantitation of the Antimicrobial Effect and its Relationships with C/MIC Ratios

Based on time-kill data, the final bacterial counts from each time-kill curve, *N*_FIN_, were recorded. In addition, for each experiment the area under each time-kill curve (AUBC) [16] was determined from the beginning of treatment to 24 h.

Imipenem/relebactam and doripenem/relebactam C/MIC relationships with AUBC observed in combined antibiotic treatments (merged data for both combinations and bacterial strains) were fitted by the sigmoid function:

*Y* = *Y*_0_ + *a*/{1 + exp[–(*x* − *x*_0_)/*b*]}, (1)

where *Y* is AUBC, *x* is log (C/MIC), *Y*_0_ and *a* are the minimal and maximal values of the AUBC, respectively, *x*_0_ is *x* corresponding to *a*/2, and *b* is a parameter reflecting sigmoidicity.

### 4.6. Statistical Analysis

The reported MIC testing data were obtained by calculation of the modal MICs. In time-kill experiments, the time-kill curves data were calculated as an arithmetic mean for two duplicate experiments, and the standard deviation (SD) was calculated.

The “AUBC-C/MIC” relationship regression analysis was performed using SigmaPlot 12.0 software (Systat Software Inc., headquartered in San Jose, CA, USA).

## 5. Conclusions

This study suggests that (1) MICs determined at a PK-based carbapenem-to-relebactam concentration ratio allow a more realistic assessment of antibiotic susceptibility in KPC-producing *K. pneumoniae* strains than MICs determined by traditional methodology and can be used to predict antibacterial effects; (2) in time-kill experiments the effects of imipenem and doripenem in the presence of relebactam are comparable.

## Figures and Tables

**Table 1:** MICs (mg/L) of imipenem and doripenem, alone or in the presence of relebactam against *K. pneumoniae*.

*K. pneumoniae* Strain	Imipenem	Imipenem in the Presence of Relebactam		Doripenem	Doripenem in the Presence of Relebactam	
		MIC_1_	MIC_2_		MIC_1_	MIC_2_
16	64	0.5	4	128	0.5	8
ATCC BAA-1902	64	0.5	1	64	0.5	2

## Data Availability

The majority of the data supporting the results of this study are located in the Supplementary Materials of this manuscript.

## Supplementary Materials

The following are available online at https://www.mdpi.com/article/10.3390/antibiotics10121520/s1, Figure S1: Time-kill curves of imipenem (circles) and doripenem (squares) against *K. pneumoniae*. Dosing regimens are indicated at each curve. Data are presented as arithmetic means. Standard deviations were not shown, as the data point difference was negligible.

## References

[1] Mansour H., Ouweini A.E.L., Chahine E.B., Karaoui L.R. (2021). Imipenem/cilastatin/relebactam: A new carbapenem β-lactamase inhibitor combination. Am. J. Health Syst. Pharm..

[2] Lapuebla A., Abdallah M., Olafisoye O., Cortes C., Urban C., Landman D., Quale J. (2015). Activity of imipenem with relebactam against Gram-negative pathogens from New York City. Antimicrob. Agents Chemother..

[3] Karlowsky J.A., Lob S.H., Kazmierczak K.M., Young K., Motyl M.R., Sahm D.F. (2020). In vitro activity of imipenem/relebactam against *Enterobacteriaceae* and *Pseudomonas aeruginosa* isolated from intraabdominal and urinary tract infection samples: SMART Surveillance United States 2015-2017. J. Glob. Antimicrob. Resist..

[4] Smith J.R., Rybak J.M., Claeys K.C. (2020). Imipenem-cilastatin-relebactam: A novel β-lactam-β-lactamase inhibitor combination for the treatment of multidrug-resistant Gram-negative infections. Pharmacotherapy.

[5] Andrei S., Droc G., Stefan G. (2019). FDA approved antibacterial drugs: 2018–2019. Discoveries.

[6] Thakare R., Dasgupta A., Chopra S. (2020). Imipenem/cilastatin sodium/relebactam fixed combination to treat urinary infections and complicated intra-abdominal bacterial infections. Drugs Today.

[7] U.S. Food and Drug Administration. https://www.fda.gov/news-events/press-announcements/fda-approves-antibiotic-treat-hospital-acquired-bacterial-pneumonia-and-ventilator-associated.

[8] Tooke C.L., Hinchliffe P., Lang P.A., Mulholland A.J., Brem J., Schofield C.J., Spencer J. (2019). Molecular basis of class A β-lactamase inhibition by relebactam. Antimicrob. Agents Chemother..

[9] Heo Y.A. (2021). Imipenem/cilastatin/relebactam: A review in Gram-negative bacterial infections. Drugs.

[10] Clinical Laboratory Standards Institute (CLSI) (2019). Methods for Dilution Antimicrobial Susceptibility Tests for Bacteria that Grow Aerobically (M07).

[11] Golikova M.V., Strukova E.N., Portnoy Y.A., Dovzhenko S.A., Kobrin M.B., Zinner S.H., Firsov A.A. (2017). Predicting effects of antibiotic combinations using MICs determined at pharmacokinetically derived concentration ratios: In vitro model studies with linezolid- and rifampicin-exposed *Staphylococcus aureus*. J. Chemother..

[12] Golikova M.V., Strukova E.N., Portnoy Y.A., Zinner S.H., Firsov A.A. (2020). Predicting the antistaphylococcal effects of daptomycin-rifampicin combinations in an in vitro dynamic model. J. Antibiot..

[13] Golikova M.V., Strukova E.N., Portnoy Y.A., Zinner S.H., Firsov A.A. (2020). Verification of a novel approach to predicting effects of antibiotic combinations: In vitro dynamic model study with daptomycin and gentamicin against *Staphylococcus aureus*. Antibiotics.

[14] Zinner S.H., Alieva K.N., Golikova M.V., Strukova E.N., Portnoy Y.A., Firsov A.A. (2021). Anti-mutant efficacy of antibiotic combinations: In vitro model studies with linezolid and daptomycin. J. Antimicrob. Chemother..

[15] Golikova M.V., Strukova E.N., Alieva K.N., Portnoy Y.A., Filimonova A.V., Zinner S.H., Firsov A.A. A pharmacokinetically-based approach to predict anti-mutant efficacy of combined doripenem and levofloxacin therapy in in vitro model studies with *Pseudomonas aeruginosa*. Proceedings of the 31th European Congress of Clinical Microbiology & Infectious Diseases.

[16] Firsov A.A., Saverino D., Ruble M., Gilbert D., Manzano B., Medeiros A.A., Zinner S.H. (1996). Predictors of effect of ampicillin-sulbactam against TEM-1 β-lactamase-producing *Escherichia coli* in an in vitro dynamic model: Enzyme activity versus MIC. Antimicrob. Agents Chemother..

[17] Clinical Laboratory Standards Institute (CLSI) (2020). Performance Standards for Antimicrobial Susceptibility Testing (M100).

[18] Keepers T.R., Gomez M., Celeri C., Nichols W.W., Krause K.M. (2014). Bactericidal activity, absence of serum effect, and time-kill kinetics of ceftazidime-avibactam against β-lactamase-producing Enterobacteriaceae and *Pseudomonas aeruginosa*. Antimicrob. Agents Chemother..

[19] Morroni G., Bressan R., Fioriti S., D’Achille G., Mingoia M., Cirioni O., Di Bella S., Piazza A., Comandatore F., Mauri C. (2021). Antimicrobial activity of aztreonam in combination with old and new β-Lactamase inhibitors against MBL and ESBL co-producing Gram-negative clinical isolates: Possible options for the treatment of complicated infections. Antibiotics.

[20] Sy S.K., Zhuang L., Beaudoin M.E., Kircher P., Tabosa M.A., Cavalcanti N.C., Grunwitz C., Pieper S., Schuck V.J., Nichols W.W. (2017). Potentiation of ceftazidime by avibactam against β-lactam-resistant *Pseudomonas aeruginosa* in an in vitro infection model. J. Antimicrob. Chemother..

[21] Montero M.M., Domene O.S., López-Causapé C., Luque S., Sorlí L., Campillo N., López M.I., Padilla E., Prim N., Angulo-Brunet A. (2021). Time-kill evaluation of antibiotic combinations containing ceftazidime-avibactam against extensively drug-resistant *Pseudomonas aeruginosa* and their potential role against ceftazidime-avibactam-resistant isolates. Microbiol. Spectr..

[22] Asempa T.E., Nicolau D.P., Kuti J.L. (2019). In vitro activity of imipenem-relebactam alone or in combination with amikacin or colistin against *Pseudomonas aeruginosa*. Antimicrob. Agents Chemother..

[23] Mueller M., de la Peña A., Derendorf H. (2004). Issues in pharmacokinetics and pharmacodynamics of anti-infective agents: Kill curves versus MIC. Antimicrob. Agents Chemother..

[24] Van der Zwaluw K., de Haan A., Pluister G.N., Bootsma H.J., de Neeling A.J., Schouls L.M. (2015). The carbapenem inactivation method (CIM), a simple and low-cost alternative for the Carba NP test to assess phenotypic carbapenemase activity in gram-negative rods. PLoS ONE.

[25] Cirillo I., Vaccaro N., Turner K., Solanki B., Natarajan J., Redman R. (2009). Pharmacokinetics, safety, and tolerability of doripenem after 0.5-, 1-, and 4-hour infusions in healthy volunteers. J. Clin. Pharmacol..

[26] Rhee E.G., Rizk M.L., Calder N., Nefliu M., Warrington S.J., Schwartz M.S., Mangin E., Boundy K., Bhagunde P., Colon-Gonzalez F. (2018). Pharmacokinetics, safety, and tolerability of single and multiple doses of relebactam, a β-lactamase inhibitor, in combination with imipenem and cilastatin in healthy participants. Antimicrob. Agents Chemother..

