# Supplementary material for: Predicting the Effects of Carbapenem/Carbapenemase Inhibitor Combinations against KPC-Producing Klebsiella pneumoniae in Time-Kill Experiments: Alternative versus Traditional Approaches to MIC Determination
Source: Antibiotics (Basel). 2021 Dec 11;10(12):1520. doi: 10.3390/antibiotics10121520 (PMC8698301; doi:10.3390/antibiotics10121520)
Supplement: Supplementary file 1 [file antibiotics-10-01520-s001.zip › antibiotics-1497693-supplementary.pdf]

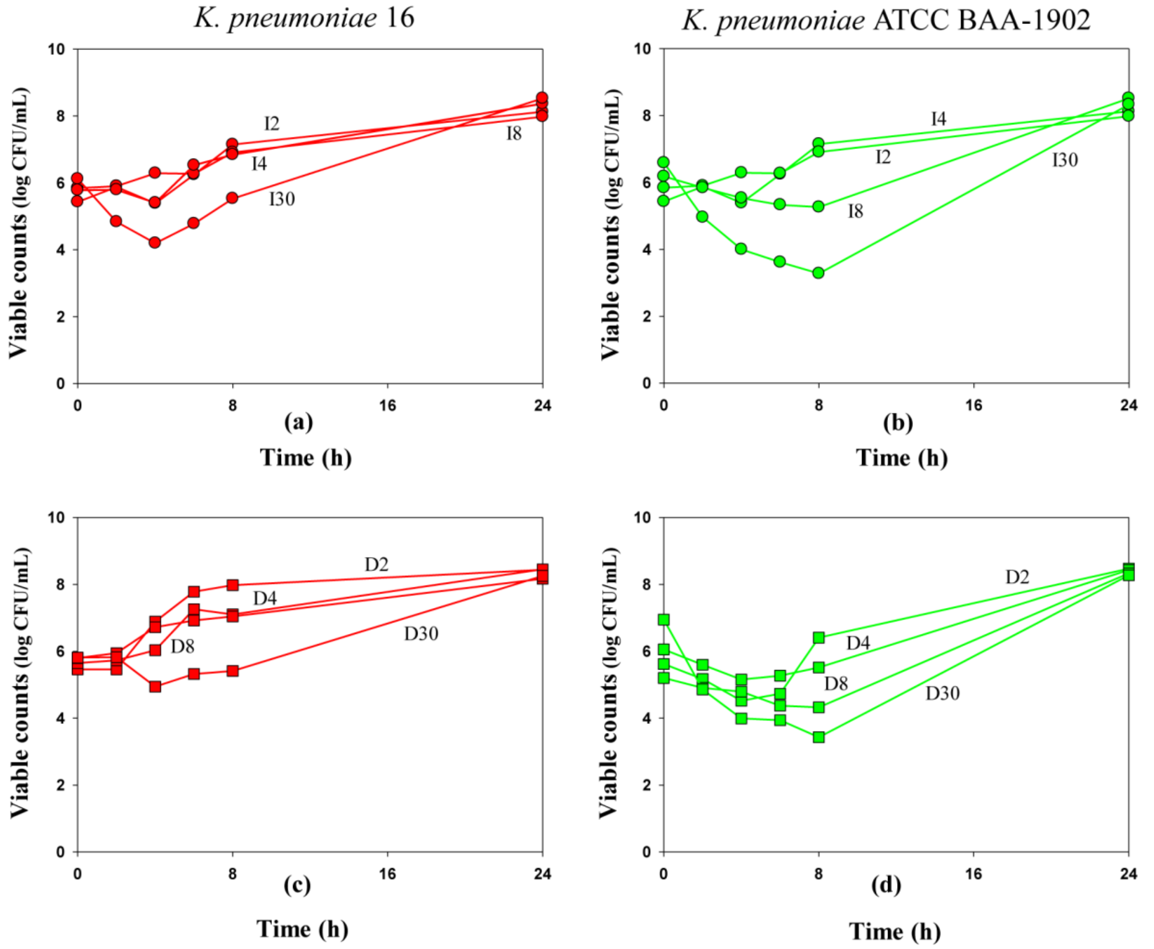

Figure S1. Time-kill curves of imipenem (circles) and doripenem (squares) against *K. pneumoniae*. Dosing regimens are indicated at each curve.
